# Comparing Miniopen and Minimally Invasive Transforaminal Interbody Fusion in Single-Level Lumbar Degeneration

**DOI:** 10.1155/2015/168384

**Published:** 2015-01-05

**Authors:** Wei-Lun Lo, Chien-Min Lin, Yi-Shian Yeh, Yu-kai Su, Yuan-Yun Tseng, Shun-Tai Yang, Jai-Wei Lin

**Affiliations:** ^1^Department of Neurosurgery, Taipei Medical University-Shuang Ho Hospital, New Taipei City, Taiwan; ^2^Keelung Hospital, Ministry of Health and Welfare, Keelung, Taiwan; ^3^Taipei Medical University, Taipei, Taiwan; ^4^University of Fukui Faculty of Medical Sciences, Fukui, Japan

## Abstract

Degenerative diseases of the lumbar spine, which are common among elderly people, cause back pain and radicular symptoms and lead to a poor quality of life. Lumbar spinal fusion is a standardized and widely accepted surgical procedure used for treating degenerative lumbar diseases; however, the classical posterior approach used in this procedure is recognized to cause vascular and neurologic damage of the lumbar muscles. Various studies have suggested that using the minimally invasive transforaminal interbody fusion (TLIF) technique provides long-term clinical outcomes comparable to those of open TLIF approaches in selected patients. In this study, we compared the perioperative and short-term advantages of miniopen, MI, and open TLIF. Compared with open TLIF, MI-TLIF and miniopen TLIF were associated with less blood loss, shorter hospital stays, and longer operative times; however, following the use of these procedures, no difference in quality of life was measured at 6 months or 1 year. Whether miniopen TLIF or MI-TLIF can replace traditional TLIF as the surgery of choice for treating degenerative lumbar deformity remains unclear, and additional studies are required for validating the safety and efficiency of these procedures.

## 1. Introduction

Degenerative lumbar diseases are common in elderly people [1]. For treating discogenic back pain and spinal instability, lumbar spinal fusion is widely used when conservative treatments fail [2–5], and numerous fusion techniques have been developed to date, such as posterior-lateral fusion (PLF) [6], posterior lumbar interbody fusion (PLIF) [7, 8], anterior lumbar interbody fusion (ALIF) [2], and transforaminal lumbar interbody fusion (TLIF) [9]. TLIF, a technique developed by Harms and Jeszenszky [10] that is commonly used today, requires little retraction of the nerve root and thecal sac but yields the benefits of circumferential fusion and maintained or regained lumbar lordosis [11, 12]. Previous studies have suggested that the traditional open posterior approach causes vascular and neurologic damage of the lumbar muscles; however, miniopen approaches can be used to avoid muscular damage and achieve lumbar fusion through noninvasive procedures [13–17]. In minimally invasive (MI) surgery, pedicle screws can be implanted percutaneously, whereas special retractors are used in the miniopen approach [18, 19]. In this study, we compared the short-term results obtained when MI-TLIF, miniopen TLIF, and traditional open TLIF were used for spinal fusions.

## 2. Materials and Methods

### 2.1. Patient Group

The participants were reviewed retrospectively and they comprised patients who had undergone single-level fusion for treatment of lumbar-spine degeneration between January 2009 and April 2012 at the Taipei Medical University system hospitals, Shuang Ho Hospital (regional hospital, New Taipei City, Taiwan) and Wan-Fang Hospital (medical center, Taipei City, Taiwan). The patients were included in this study if they (1) had degenerative discopathy or spinal instability, (2) had no previous lumbar-spine surgery, and (3) had undergone single-level fusion.

### 2.2. Miniopen TLIF

**Figure 1:**
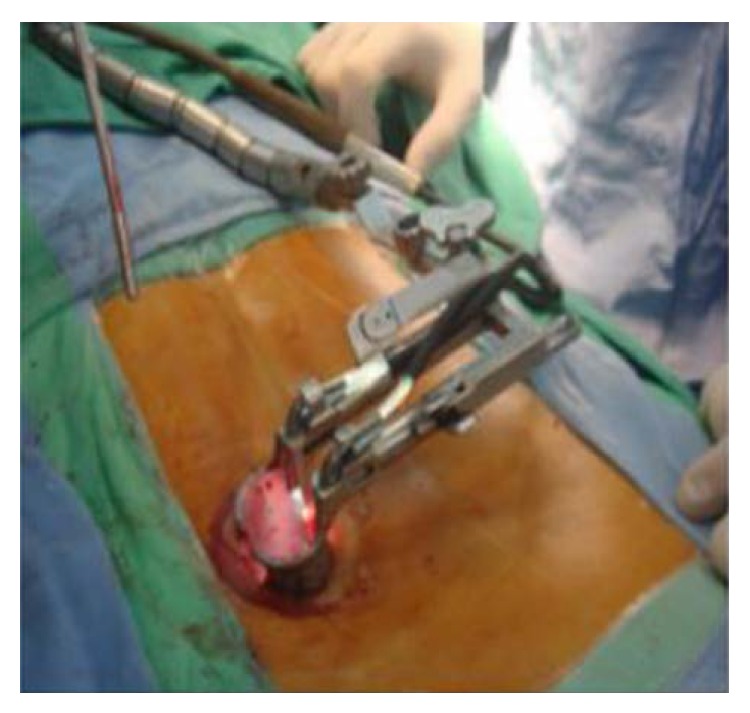
Miniopen TLIF with Mast Quadrant system.

**Figure 2:**
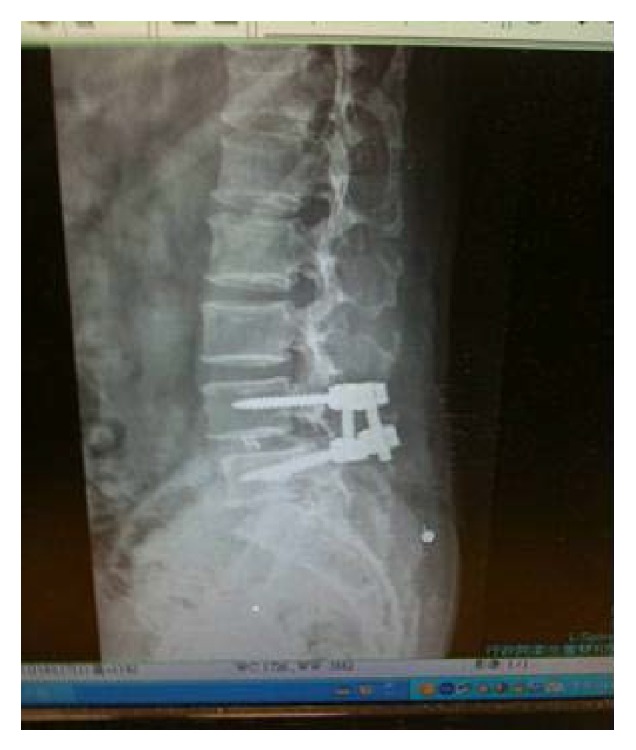
Postoperative image after miniopen TLIF.

We used the Mast Quadrant system and the Wiltse paraspinal approach on the lumbar spine. The patients were positioned prone and provided with general anesthesia. Using fluoroscopic guidance, we determined the site and length of the skin incision and subsequently made a 2-3 cm skin incision over the paramedian region, 2.5 cm lateral to the medial back line. After dissecting the superior and deep fascia, we inserted a 20-gauge needle at the operative site and then longitudinally separated the sacrospinalis muscle between its multifidus and longissimus. Next, a series of tubular dilators was advanced to produce an exposure sufficiently large to perform the procedure through an appropriately sized tube. A tube was inserted over the dilators, placed firmly until flush with the bony anatomy, and then locked in place by using a flexible arm. Subsequently, the dilators were removed to establish a tubular operative corridor. After this tube is in place, various procedures can be performed (Figure 1). The transmuscular approach is used for the purpose of performing laminotomy, medial facetectomy, foraminotomy, discectomy, TLIF, or pedicle-screw insertion. In order to achieve fusion, we inserted the Capstone cage as the stent (Figure 2). After the operation, the dura was covered using a small amount of subcutaneous fat to prevent adhesion.

### 2.3. Minimally Invasive TLIF

We used a Sextant system and the Wiltse paraspinal approach to perform MI-TLIF [20]. The procedure was performed at the fusion by using the same process used in the miniopen TLIF. When using the MI-TLIF method, a screw was placed only on the contralateral site. This operation was performed percutaneously and it yielded a small wound and surgical field. After patients were placed under general anesthesia and positioned prone, we used fluoroscopic guidance to mark an appropriate spine level and then made a 3 cm incision 4.5 cm from the midline. A k-wire was targeted at the bony complex at the surgical level and a series of dilators was passed consecutively to split the muscle fibers; proper orientation of dilator was confirmed using fluoroscopic imaging. A working channel was placed, the dilators were removed, and the channel was secured appropriately in order to visualize the medial portion of the facet and inferior lamina. The fusion and decompression procedures were subsequently performed using the same methods as those used in miniopen TLIF. The decompression procedure was completed under a microscope.

### 2.4. Data Analysis

We determined the patients' age, blood loss, surgery time, and the timing of getting out of bed following the operation. The pain scores, the length of hospital stay, the number of infections, and cerebrospinal fluid (CSF) leakage were also recorded.

## 3. Results

Among the participants, 36 patients underwent MI-TLIF, 817 underwent miniopen TLIF, and 120 underwent traditional open TLIF (Tables 1 and 2). The recorded results were the following. Open-TLIF patients' results were as follows: the mean age was 57.2 years, the estimated blood loss was 250 cc, the average surgery time was 92 min, the mean time until leaving the bed was 3.8 days, the mean pain score on the second day following the operation was 7.5, and the mean hospital stay was 10.2 days; 2 patients (1.6%) developed infections, and 3/120 (5%) patients exhibited CSF leakage. Miniopen-TLIF patients' results were as follows: the mean age was 56.4 years, the estimated blood loss was 130 cc, the average surgery time was 108 min, the mean time until leaving the bed was 2.2 days, the mean pain score on the second day following the operation was 2.8, and the mean hospital stay was 2.8 days; 5/817 (0.6%) patients exhibited CSF leakage, and no patients developed postoperative infections. MI-TLIF patients' results were as follows: the mean age was 53.5 years, the estimated blood loss was 120 cc, the average surgery time was 138 min, the mean time until leaving the bed was 1.5 days, the mean pain score on the second day following the operation was 2.6, and the mean hospital stay was 5.8 days, and no patients exhibited postoperative infections or CSF leakage.

## 4. Discussion

Various fusion techniques can be used for treating patients who exhibit degenerative lumbar-spine diseases. After fusing unstable segments, the mechanical back pain that results from pars defects or facet arthropathy can be decreased, which leads to favorable functional outcomes [21, 22]. PLF was the first fusion technique developed, and it typically requires an autologous bone graft harvested from a site such as the iliac crest. However, after interbody fusion techniques were developed, PLF has not been used because it caused pain at the donor site of the bone graft and yielded lower fusion rates as compared with interbody fusions [23–25]. The primary routes of lumbar interbody fusion are anterior, posterior, and transforaminal. ALIF is used infrequently because it requires a surgical technique that most surgeons are unfamiliar with and because it yields high injury rates in vascular structures, the sympathetic chain, and the hypogastric plexus [26–28]. PLIF was the first interbody fusion technique developed and it is now commonly applied [2, 29]; however, root traction can cause complications such as neurogenic pain or nerve damage when PLIF is used. Moreover, PLIF is typically limited to the L3–S1 discs because it increases the risk of damage to the conus medullaris and cauda equina [2].

In 1998, Deguchi et al. developed the TLIF technique, in which an interbody cage packed with a bone graft is inserted through a transforaminal route [7]. This technique requires little retraction of the nerve root and the thecal sac and yields the benefits of circumferential fusion and maintained or regained lumbar lordosis. Currently, TLIF is widely used in lumbar spinal fusions. Previously, the traditional open posterior midline approach was reported to cause vascular and neurologic damage of the lumbar muscles; thus, the use of a paramedian (Wiltse) approach yielded advantages over the midline approach, such as comparatively less damage to the multifidus muscle and decreased postoperative analgesic requirements [30]. Therefore, miniopen TLIF and MI-TLIF were developed, and numerous studies compared traditional and MI-TLIF surgeries or miniopen and traditional TLIF surgeries [13–16, 31, 32]. Compared with open TLIF, MI-TLIF required less dissection of the psoas muscle as a result of morbidity and caused less intraoperative blood loss and shorter hospital stays [33]. However, the problems associated with MI-TLF are increased operative times and exposure to fluoroscopy radiation and a high learning curve for surgeons. Furthermore, certain studies have reported that open TLIF and MI-TLIF yield similar long-term outcomes [34] and produce similar rates of complications such as graft malposition and CSF leakage [18, 34]. Furthermore, the use of MI-TLIF appears to slightly increase the rate of nerve-root damage.

In this study, we compared the outcomes of traditional open TLIF, miniopen TLIF, and MI-TLIF when these procedures were used for treating single-level lumbar degeneration. The results indicated that miniopen TLIF yielded outcomes that were superior to those of traditional open TLIF in terms of operative blood loss, postoperative pain scores, time at which patients were out of bed, timing of getting out of bed after the operation, infection rate, and CSF leakage. Because the facet joint is directly approached when using miniopen TLIF, little muscle dissection or bone removal is required for the spinous process or lamina; this reduces the rates of death space, blood clots accumulation, and tissue fluid accumulation. Therefore, the infection rate is lower in the case of miniopen TLIF than in that of traditional open TLIF. In patients who undergo reoperation, surgical difficulty is decreased because a small fat graft can be used to cover the dura, and the operation plane can be readily exposed. A comparison of the short-term results of miniopen TLIF and MI-TLIF yielded similar results in terms of blood loss, the timing of getting out of bed after operation, pain scores, hospital stay, number of infections, and CSF leakage. Surgery time was the shortest in the case of the miniopen TLIF procedure, potentially because of enhanced surgical exposure. Furthermore, in miniopen TLIF, bilateral decompression can be performed when severe stenosis is located preoperatively. Certain studies have suggested that the disadvantages of MI-TLIF include the anatomical disorientation caused by unexposed landmarks, small working areas that require long, bayoneted instruments, and the high learning curve [35–37]. However, a learning curve is a feature of all applications of MI surgeries, because these provide little surgical exposure and require distinct instruments when compared with other surgeries. Therefore, compared with MI-TLIF, miniopen TLIF offers greater surgical exposure and a lower learning curve but yields similar short-term results.

## 5. Conclusions

MI-TLIF yields the optimal short-term results in the case of lumbar-spine surgery; however, this procedure is lengthy and takes a long time to learn. By comparison, miniopen TLIF yields greater surgical exposure and requires a shorter learning period and surgery time; however, patients who exhibit multilevel degeneration might experience increased muscle dissection and ligament injury when miniopen TLIF is used. Further investigation must be conducted.

## Figures and Tables

**Table 1:** Demographic data of each surgical approach.

	Traditional open TLIF	Miniopen TLIF	Minimally invasive TLIF
Number	120	817	36
Mean age	57.2	56.4	53.5
Estimated blood loss (mL)	250	130	120
Surgery time (min)	92	108	138
Timing of getting out of bed (day)	3.8	2.2	1.5

**Table 2:** Results and complications of each approach method.

	Traditional open T LIF	Miniopen TLIF	Minimally invasive TLIF
Mean VAS (pain score) on second day after surgery	7.5	2.8	2.5
Hospital stay (day)	10.2	6.3	5.8
Postoperative infection: number (percentage)	2 (1.6%)	0 (0%)	0 (0%)
CSF leakage: number (percentage)	3 (5%)	5 (0.6%)	0 (0%)
